# Emotion Processing by ERP Combined with Development and Plasticity

**DOI:** 10.1155/2017/5282670

**Published:** 2017-07-31

**Authors:** Rui Ding, Ping Li, Wei Wang, Wenbo Luo

**Affiliations:** ^1^Research Center of Brain and Cognitive Neuroscience, Liaoning Normal University, Dalian 116029, China; ^2^Laboratory of Cognition and Mental Health, Chongqing University of Arts and Sciences, Chongqing 402160, China

## Abstract

Emotions important for survival and social interaction have received wide and deep investigations. The application of the fMRI technique into emotion processing has obtained overwhelming achievements with respect to the localization of emotion processes. The ERP method, which possesses highly temporal resolution compared to fMRI, can be employed to investigate the time course of emotion processing. The emotional modulation of the ERP component has been verified across numerous researches. Emotions, described as dynamically developing along with the growing age, have the possibility to be enhanced through learning (or training) or to be damaged due to disturbances in growth, which is underlain by the neural plasticity of emotion-relevant nervous systems. And mood disorders with typical symptoms of emotion discordance probably have been caused by the dysfunctional neural plasticity.

## 1. Introduction

Emotion, as a form of information delivery, serves as a critical role in human survival and social communication, from which humans can receive the signals of threats and messages individuals would use to correspond in correct ways. Thus, the emotion processing has been attracting numerous researchers into the exploration of it (for more details, see LeDoux et al. [1], Phan et al. [2], Russel [3], Ochsner and Gross [4], Lindquist et al. [5], and Olofsson et al. [6]). Especially, the application of event-related potential (ERP) and functional magnetic resonance imaging (fMRI) techniques into psychological and neurological science enables the assessment of affective responses with millisecond temporary resolution and high spatial resolution, respectively, with increasing emergence of studies for emotion processing. Through functional imaging, studies have depicted extensive emotion-relevant brain networks involved in emotion processing in human subjects, regions of which include traditional visual cortices (e.g., the face-selective fusiform gyrus, superior temporal gyrus) [7–10], amygdala [11–13], orbitofrontal cortex [9, 14, 15], right frontal-parietal cortices [9, 16], somatosensory related cortices (i.e., insula cortex) [17, 18], and basal ganglia [19–21] and maybe auditory cortices for processing emotion-related prosy [22, 23]. Likewise, the electrophysiological studies for processing affective stimuli have offered us various insights into emotion processes from the temporal dimension. An early study by Begleiter et al. in late 1960s already demonstrated the amplitude variability with emotion category, in which stimuli conditioned by unpleasant words elicited larger peak-to-peak amplitude, compared to those associated with neutral and pleasant words [6]. More recently, the ERP studies have consistently confirmed the ERP components which are sensitive to processing the emotional stimuli, such as N100, P100, N170, vertex positive potentials (VPP), N250, N300, P300, late positive potentials (LPP; or as late positive complex (LPC)), and early posterior negativity (EPN) [24–28]. However, the magnificent achievements in spatially localizing the emotion processing, as well as the enthusiasm for assessing emotion processes by means of fMRI, contrast vividly with modest progress in the temporal course of processing emotion.

fMRI using BOLD (blood oxygenation level-dependent effect) contrast, since firstly applied into exploring functions of human cortex in 1992 [29, 30], has been receiving welcome from neuroscience and psychological researchers. fMRI technique with its high spatial resolution and noninvasiveness has exhibited an immense advantage in exploring the relationship between neuroanatomy and cognitive processes, compared to other functional imaging methods. The subsequent event-related design fMRI approach [31, 32], conforming to the logic ERP studies adopted, allows randomly the presentation of experimental trials and controlled trials, which creates a higher level of assurance that experiment hypothesis reflects the differences between contrasts (i.e., conditions) than the block design. Additionally, functional connectivity (FC) analytical methods [33, 34] can be applied to explore whether changes of certain brain region activity contact with others or not, and in that way, we can confirm the cognition and emotion-relevant neural network. Using the FC method, researchers have investigated and endeavored to identify the emotion-relevant network in healthy individuals and in mental disorder populations [5, 35–37]. Still, the shortcomings exist in fMRI studies. Unlike ERP directly measuring the evoked potentials by brain activity, the fMRI method utilizes the hemodynamic property of the brain and surveys the indirect metabolism signals induced by cerebral activities, leading to delayed rising of BOLD with several seconds after stimulus presentation or cognitive manipulation (i.e., the temporal resolution of fMRI is lower than that of ERP). Despite the terrible spatial resolution, the ERP technique allows researchers to assess emotion processing with millisecond temporal resolution. Thus, ERP provides us with an excellent tool to look into the temporal course of emotion processing, which fMRI lays its deficits on. Given the shortcomings of fMRI and the modest progress in the time course of emotion processing, it is imperative to further investigate when experience evoked by emotional stimulus affects event-related potentials and even deep into how emotion signals are processed in the brain through the combination of the fMRI and ERP methods.

Emotion, as acknowledged, is more than the statical innate ability to deliver information about survival and threats, but also a competence of developmental attributes, by which ones can respond to diverse situations with increasing properly emotional-expressive behaviors [38]. Thus, emotion to be developmentally investigated is what we should consider when exploring the mechanism for emotion processing. The longitudinal studies using fMRI have verified the brain regions of neural plasticity for emotion processes [39–41]. In addition to neural imaging method, ERP could be utilized to characterize the temporal changes of emotion processing along with the growing of individuals. It is legitimately expected that more refined developments in emotion-relevant areas could be localized through the combination of ERP and fMRI. Moreover, the ERP technique can induce and measure the long-term potentiation (LTP), as a correlate of neural plasticity, in the human visual and auditory cortex, and provide a noninvasive instrument to assess LTP-associated plasticity in human beings [42, 43]. From another point of view, the disturbances of such neural plasticity contribute, at least partly, to the generation of mental disorders of emotional deficits, for instance mood disorders and schizophrenia [44]. The researches using the ERP technique endeavored to assess the impaired neural plasticity in patients with these disorders and obtained significant results [45, 46]. It is therefore feasible and required to probe the neural plasticity for emotional development by means of ERP.

To sum up the above arguments, the present review collected the emotional ERP studies from the recent 10 years (2007–2016) and integrated the emotional effects on ERP components, combined with the review of emotional development studies and studies regarding neuropsychiatric disorders for emotional neural plasticity. Additionally, this review discusses the issues that may contribute to emotional effects on ERPs, including methodology, stimuli characteristics, and emotion categories.

## 2. Exploring Emotion Processing by Means of the ERP Technique

### 2.1. Categories and Dimensions of Emotion

According to the studies by Osgood et al., Russel et al., and Lang's discourse on emotional dimensions [47–50], emotion could be viewed as a continuum of two dimensions, namely, valence and arousal. The valence dimension refers to the degree in which individuals feel pleasant or unpleasant, and arousal dimension denotes the subjective state of feeling activated or deactivated (i.e., denoting the intensity of internal emotion response) [51]. Based on the cross-culture study of facial expressions, Ekman and his colleagues proposed that happy, sad, fear, anger, disgust, and surprise represented the six basic affective states of emotion [52, 53]. The six basic affective states could be placed into the circumplex model of affect by Russel, with the respective values of valence and arousal [48]. Lang et al. hold the position that the valence viewed as a manifestation of motivational systems in the brain and the arousal denoting the intensity of the motivational systems would to some extent interact with each other [54]. Thus, the level of arousal should be controlled when valence effect is the research target. Apart from the six basic emotional states, one class of emotions which we called the social emotions plays a critical role in social interactions. Social emotions including pride, guilt, jealousy, shame, and embarrassment develop later than the above basic emotions and are more dependent on social context [55].

### 2.2. Issues Accompanied with ERP Application into Emotion Study

ERP, as an experimental tool to temporally explore cerebrum processing, is sensitive not only to properties of stimulus presented to subjects but also to mental states of subjects [56, 57]. Especially, when investigating affective processing, it is critical to avoid the confounding between stimulus and emotional effects on ERP components. Additionally, ERP extracts event-related signals from the background rhythms of brain electric activity [56], and in consequence, the methodology for ERP studies of emotion processing requires considerations. As the above review suggests, affective states correlate with a distributed network of brain areas, with differential cerebral localizations responding to different emotions. Reflected in ERPs, various emotions might exert distinct temporal dynamics. In conclusion, emotion categories as well as methodological considerations and stimuli characteristics will be discussed in the subsections below to elucidate how to elicit reliable emotional response/experience and how to ensure the real effects of emotion on ERPs.

#### 2.2.1. Methodological Considerations

Most ERP studies of emotion processing generally employ the facial affective pictures as stimuli to elicit emotional response/experience, and these affective stimuli were mainly extracted from the standardized datasets, such as the International Affective Picture System (IPAS) and Chinese Facial Affective Picture System (CFAPS) [58–60]. Take IPAS as an example, the system received ratings of valence and arousal from subjects during free view [58, 61]. ERP studies however are overtly distinct from free view situations where the level of arousal for identical emotional stimulus differed from the former, especially when the experiments confine the stimulus duration/presentation velocity [62–64]. It is consequently essential to execute the prudent and rigorous manipulations of the two emotional dimensions, namely, valence and arousal, in which arousal influence could be measured and analyzed to disentangle effects of valence and arousal on ERPs. Available adoptions like subjective ratings of valence and arousal within the ERP programs could be applied. Or like Aguado et al. and Utama et al. implemented in their studies [65, 66], the same population of subjects was requested to rate the intensity of stimuli in psychological experiment followed by the electrophysiological experiment with selected stimuli based on previous ratings and with similar program of evaluating the intensity level as in the former experiment.

In order to obtain the reliable ERP components with high signal-to-noise ratio, a large scale of trials constituted by visual or auditory stimuli is needed [56]. In ERP studies of emotion processing, the trials of stimuli should be repeated for enough times to insure reliable and steady ERP components extracted from electroencephalographical (EEG) signals. However, the repeated experience of an identical visual stimulus would suppress the intensity of stimulus-related neuronal responses, namely, repetition suppression effect [67]. Fiebach et al. and Matsumoto et al. explored the ERP evidence of whether repetition suppression phenomenon existed during processing the repeated stimuli and found that N400 reduced significantly when stimulus-related information was repeatedly presented to subjects [68, 69]. Thus, using ERP to research emotion processing should endeavor to avoid the decreasing response to affective stimuli, which to some extent contradicts with the practice that repetition of stimuli-involved trials enables reliable and stable ERPs. Carefully, experimental design, with random and pseudorandom allocation of sequence of the emotional stimuli as well as trials, is necessarily considered to lower the subjects' familiarity with stimuli, especially faces and words. Moreover, just like Olofsson et al. proposed [6], researchers can assess the repetition effect evoked by repeated stimuli, with the purpose to evaluate whether repetition of stimuli changes over valence and arousal conditions.

As we reviewed above, emotion processing involves a complex and distributed network of cerebral regions. Consequently, assessing the spatial distribution of emotion-evoked ERPs is necessary. With the increasing complexity of ERP datasets obtained by high-density electrode arrays, the analysis method like principle component analysis (PCA) and independent component analysis (ICA) with more reliability can be employed into analyzing the ERP datasets obtained by high-density device. Meanwhile, it is partly helpful for circumventing certain restrictions of conventional ERP analytical methods, like insensitivity to brain activity indicated by low ERP signal and reference dependence (i.e., ERP amplitude varies with selections of reference electrodes) [70, 71]. Another advanced method called low-resolution brain electromagnetic tomography (LORETA) could combine with a head model constructed by the previous scanned MRI structural images to assess the spatial dynamics of emotion processing [56, 72, 73].

#### 2.2.2. Stimulus Considerations

Emotion, as a systematical behavior with complex brain activities, incorporates various expressing modalities. As Adolphs reviewed, individuals can recognize emotions from multiple sensory modalities, including facial expressions, emotional prosody, and body expressions, or from the integration of multiple modalities [74]. When studying emotion processing, systematical investigation of emotion recognition from multiple modalities cannot be ignored, where temporal dynamics of emotion processes probably are affected by modalities due to the differential sensory pathways. However, the ERP studies for emotion processing mainly employed the facial expressions as emotion-eliciting stimuli. Compared with the extensive investigation into emotion triggered by visual modality, the ERP studies regarding affective processing seldom involve other modalities, while utilizing ERP into emotion response/experience elicited by vocal, bodily, and touching stimuli earned significant results [75–77]. What we elaborated above is for the one hand, and on the other hand, the integrality of emotion perception should be taken into account. Simultaneously, perception of emotional information comes from multiple sensory modalities, that is, individuals perceive and process concurrent input of affective cues including facial expressions, voice, and body expressions. And this concurrent input of multimodal information generally enhances the emotion perception and recognition [78, 79]. The ERP studies therefore should further involve exploration into the temporal integration of multimodal affective info, which even though have already found the interaction between facial and body expressions as well as between emotional prosody and body expressions [79, 80]. Moreover, integration of multisensory modalities adheres to demand for improving the ecological validity.

The reason why ERP researchers attended less to emotion-expressing modalities of voice, body, and touching may lead to less compatibility with complex physical properties of stimulus. ERP waves are sensitive to physical attributes of stimulus and changes of environment [56, 81]. And such fact necessitates the premeditation of controlling and maybe evaluating stimulus physical properties and environmental variables. In addition, a certain number of researches probed into whether the physical properties of stimulus would affect the effects of emotional dimensions on ERP components, and found featural size, color, spatial frequency, and complexity as confounding factors in explaining the emotional effects on ERPs [82–85]. Thus, ERP studies of emotion processing have to strictly control the physical variables or to assess the overall complexity towards subjects as Carretié et al. performed in their study [86]. Numerous studies have shed light on the interaction and integration between emotion and sensory processing (e.g., emotional stimuli gain more rapidly processing relative to nonemotional ones) [87], yet few are known about how perceptual information interacts with processing emotional stimuli. The relationship between emotion processing and selective attention towards more salient stimulus will enlighten us in exploring the mechanism by which the stimulus physical properties modulate emotional response/experience [88].

Various affective systems have been developed for emotion elicitation and measurement based on differed cultural populations which been validated, such as IAPS [58], CFAPS [59], the Geneva Affective Picture Database (GAPED) [89], the Karolinska Directed Emotional Faces (KDEF) [90], NimStim Face Stimulus Set [91] for visual elicitation, and the International Affective Digital Sounds (IADS) [92], the Montreal Affective Voices (MAV) [93], and the Acoustic Profiles in Vocal Emotion Expression [94] for auditory elicitation. According to the discrete emotional model by Ekman [53], the basic emotions are ubiquitous through all cultures. Nonetheless, the cultural differences were still observed in recognition of emotions [95–97]. ERP study by Hot et al. found the electrophysiological evidence for cultural differences during emotional processing, in which the later components' amplitudes significantly decreased for Japanese subjects relative to French ones [98]. The databases based on the specifically cultural population necessitate the consideration of applicability towards current subjects. For further assurance of stability in evaluating valence/arousal, pilot study could be performed to have subjects voluntarily assess the dimensions. Another way to select the stimuli that we can produce on our own however arises another trouble that these made-up stimuli receive no validations and standardizations. The real-life emotion perception and production is a continuing affective processing. Studies regarding the sustained processing of emotion ordinarily employed the film clips as eliciting stimuli, instead of static pictures. This practice could supply individuals with more real-life accordant scenarios and more authentic emotional response/experience. The film clips databases have therefore been exploited to improve ecological validity. The current existed databases like IAPS and GAPED have been already added with film clips [61, 89]. It is worth noting that most of film clips databases could elicit discrete affective states [99]. On the other hand, IAPS validated the validity of film clips in eliciting emotional experience measured by valence and arousal, and so as the Emotional Movie Database (EMDB) [61, 99]. Although low numbers of ERP studies were reported with the emotion elicitation by film clips, yet like Chwilla et al. performed [100], reasonable and cautious experimental design would enable such method possible in ERP studies.

#### 2.2.3. Emotion Categories

Most of the studies using ERP into emotion processing targeted the six basic emotional states, especially happy, sad, and fear, which is independent of social concerns (e.g., situation where emotions happen). However, emotion is generally, as acknowledged, the result of social events and interacts with social processes [101–103], which cannot be overlooked when considering conceptual emotions from the perspective of functionalism [104]. The researches need considerations of socially elicited emotions, especially when we take development of emotion processing into consideration. Some theorists, from another point of view, distinguish the two groups of elicited emotions into separate categories [105, 106]. The social emotions defined as a specific subset of emotions, compared to basic emotions, commonly incorporate envy, jealousy, shame, guilty, embarrassment, admiration, and so on. Norris et al. examined and verified the significant interaction between social and emotional processes [107], and the recent ERP study regarding the emotion recognition investigated and showed the significant difference of slow positive wave (SPW) on recognizing happiness and pride [108]. Even so, the social emotional processes are still much less well investigated than the basic emotional processing, possibly due to its inherent complexity, subjectivity, and prolonged experience [109]. And it remains to be resolved in the future.

#### 2.2.4. Other Considerations

Based on James' peripheral perception theory and basic emotion theory by Ekman et al. [52], the emotion response is a systematical behavior incorporating psychological experience as well as physiological reflections which could be indexed by heart rate (HR), skin conductance level (SCL), respiratory rate (RR), and so on. And these indexes have been validated to be correlated with assessing the affective valence and arousal ratings [110–113]. Moreover, the physiological patterns accompanied with emotion response/experience have been utilized to classify the emotion categories, with high accuracy even in identifying the emotion of unknown individuals [114]. On the other hand, the physiological indexes, like HR, SCL, and RR, would also affect the electrophysiological signals, leading to interference with ERPs. Altogether, it is worth a try to measure these indexes and evaluate the influence on ERPs mirroring emotional processing other than removing and correcting artifacts based on certain algorithms (e.g., ICA).

## 3. Emotion Processing Revealed by ERPs

Numerous studies have been performed to answer the anatomical questions about emotion processing (for review, see Lindquist et al. and Dricu and Frühholz [5, 115]), especially for the six basic emotional states. However, answering the question like how cerebrum processes the affective information not only needs the studies of spatial properties but also needs investigating the temporal dynamics of processing emotion. With high temporal resolution, the ERP technique would perform surpassingly in temporally characterizing the emotion processes, which will improve the understanding of when emotion recognition happens. And characterizing the temporal order of emotional ERPs would help to further investigate and speculate the specific ERP components responding to the specific emotions' recognition. It is therefore in demand, for further researching emotion processing temporally, to integrate the findings of ERP studies on emotion. In the following section, we would collect emotion-relevant studies using ERP and discourse the emotional effects on ERP components. Given the previous review by Olofsson et al. based on literatures of 1966–2007, we searched the studies published during 2007–2016. Based on past ERP studies and their own works, Luo et al. proposed the three-stage model to describe the time course of emotion processing. The model holds that the ERP components have differential preference for distinct emotional facial expressions [25]. And the various ERP components could contribute to emotion distinguishing in three stages: the first stage for automatic but coarse processing (N100 and P100), the second stage for distinguishing emotional and neutral facial expressions (N170 and VPP), and the third stage for distinguishing various emotional facial expressions (N300 and P300). The model has been extended to processing emotional words by Zhang et al. [116]. The following review mainly focus on the above ERP components with the additional EPN and LPP.

### 3.1. P100 and N100

P100 with an onset latency of 60–80 ms is a positive-direction component which peaks at around 100–130 ms after stimulus onset and generally is detected at the parieto-occipital electrodes. There exist recently ERP studies investigating the modulation of P100 by emotionality including valence and arousal factors. Luo et al. employed rapid serial visual presentation (RSVP) paradigm to explore the electrophysiological correlates of facial expression processing as well as the attentional blink effects on emotional facial expressions and found that P100 was significantly affected by facial emotions (i.e., fearful facial expressions elicited higher P100 than happy and neutral ones) [25]. And the results are consistent with the previous study by Utama et al. and the subsequent study by Aguado et al. [65, 66]. The latter two studies, respectively, manipulated the arousal and valence of stimuli to discuss whether the two dimensions correlated with P100 and returned with results of significant correlations for valence yet insignificant for arousal. Some ERP studies meanwhile applied other emotion-eliciting stimuli which include emotional words, emotional sentences, and emotional scenes [117–119]. Especially worth to note, the study by Rochas et al. combined EEG and transcranial magnetic stimulation (TMS), in which TMS was used to interfere the word-reception region, at the time when emotion significance was processed (i.e., between 70 and 200 ms). And the results showed slower detection of emotional words as compared to neutral words [117]. Moreover, Conty et al. assessed the interaction among gaze direction, body gesture, and facial expressions and also found the main effect of anger expressions on P100 activity except from the significant interaction [120]. Thus, based on the above sum-up, it is probably concluded that emotion processing starts at around 100 ms after stimulus onset but no integration of emotion significance and other social information.

For N100 considered as a sensory component, the work by Luo et al. indicated the significant emotion modulation of N100, in which fearful faces elicited larger N100 amplitudes than happy and neutral faces [25]. The results are in accordance with the previous studies [121, 122] and with the theoretical consideration that unpleasant stimuli can more easily capture the attention resources relative to pleasant and neutral ones [123]. Another study by Pell et al. with nonlinguistic vocals involved found that happy vocal stimuli significantly reduced the N100 latencies as compared to sad and anger stimuli but no significant main effect of emotion on N100 amplitudes [124], which may suggest the differences between processing emotional faces and emotional vocals. Some of the researches with emotional words also test the N100 differences elicited by emotionality, few of which reported significant effect of emotions on N100 amplitudes and latencies [116, 125]. And regarding the reason, this may lead to the visual fields of word presentation [126, 127]. Additionally, the integration of emotion modalities to some extent has been explored. Jessen and Kortz combined vocal, facial, and body expressions into the experimental paradigm and showed us the interaction between auditory and visual modalities reflected by N100 (i.e., N100 amplitudes were elicited smaller at audiovisual condition compared to unimodal condition) [79]. Combined with the unanimous findings about emotional effects on N100, conclusion could be made that N100 is probably influenced by complex factors to be dissociated and maybe indicate the interaction among various modalities.

### 3.2. N170 and VPP

N170 is a negative-going ERP component detected at the lateral occipitotemporal electrodes, which peaks around 170 ms after stimulus onset. This component has been found to be sensitive to face stimuli rather than nonface stimuli [128]. Most ERP studies about emotion processing involved the analysis of N170 component. In study by Luo et al., experimenters recorded the electroencephalographic signals locked to emotion response and obtained the significant emotional modulation of N170, in which happy and fearful facial expressions elicited larger N170 amplitudes and shorter latencies than the neutral faces [25]. This study employing the RSVP also found the significant differentiation between fearful and happy expressions by N170, which indicated that N170 may contribute to distinguishing the emotional facial expressions. Such findings received supports from other studies [129–132]. Moreover, another supporting study with magnetoencephalography (MEG), in which a sphere head model was scanned by MRI and fitted to the inner skull surface, in the fusiform gyrus, showed larger amplitude elicited by fearful expressions than by neutral faces at 170 ms [133]. However, some researches yielded contradicting outcomes. Like Leppänen et al., they manipulated the intensities of fearful and happy emotional expressions and found no significant emotion effect on N170 amplitudes [134], which other studies agreed with [135–137]. A meta-analysis included 57 emotion studies and calculated the overall effect size describing N170 responses to emotional faces, which exhibited larger N170 amplitudes elicited by anger, fear, and happy facial expressions relative to neutral faces [138]. The meta-analysis also investigated the moderators that may affect N170 differentiation between emotional faces and neutral ones and suggested that N170 was sensitive to unattended stimuli and was a subject to the reference electrodes. To sum up, N170 as a valuable instrument can be effectively applied to study the neural processing of emotion, but modulatory factors need further consideration and investigation.

VPP is a positive component with a peak latency similar to that of N170 and is detected at the frontocentral electrodes. Rossion et al. verified VPP's sensitivity to face stimuli processing other than N170 [128]. The ERP researches regarding the N170 component generally report the analytical results of the VPP component. Consistent with the previous studies [139–141], Luo et al. obtained the significant emotional effect on VPP amplitudes as well as on VPP latencies, in which happy and fearful faces elicited larger VPP amplitudes than neutral facial expressions. Contrasted with the facts that few studies included in this review reported the significant emotional modulation of VPP latencies (or chose not to report the results about VPP latencies), Luo et al. also showed that shorter latencies were elicited by fearful facial expressions than by happy and neutral faces [25]. The subsequent studies additionally provided the support for findings obtained by Luo et al. [142–144]. It is evidenced that early processing indicated by N170/VPP occurs in discriminating fearful/happy facial expressions and neutral faces [145]. Other than such point of view, the studies contrasting positive and negative emotions suggested the possible differentiation between positive and negative facial expressions by VPP. For instance, Willis and Rburke involved anger and happy faces in the study and detected the significant ERP difference between two emotions with larger VPP amplitudes elicited by angry blocks than by happy blocks [144]. And in another study, the disgust facial expressions evoked larger VPP amplitudes than the happy faces [142]. Agreement therefore could be made that negative facial expressions receive perhaps more early processing as compared to other facial expressions.

### 3.3. EPN

EPN, namely, early posterior negativity, peaks at 210–350 ms with a topographical distribution over occipitotemporal sites. The EPN effect for emotionality has been normally reported in studies using emotional faces and words to elicit emotion response/experience. The very recent study by Calvo et al. assessed the emotion effect on ERPs with the emotion-eliciting stimuli of whole faces and half faces employed, in which the EPN amplitudes for angry expressions were larger than those elicited by other facial expressions in the right hemisphere and were larger than those for neutral faces in the left hemisphere, but the above effects were only seen in whole face condition [146]. And the results indicate that the early lateralized processing (e.g., brain activity reflected by N170 and VPP) possibly encodes the holistic features of emotion. The work by Zhang et al. obtained significantly larger EPN amplitudes for positive and negative words than for neutral words. In this study, hemisphere effect on EPN amplitudes was observed as well, with more negative-going EPN for emotional words than the neutral ones in the left hemisphere [116], which is consistent with the abovementioned study. But unlike the study by Calvo et al., Zhang et al. reported no significant difference of EPN amplitudes between negative and positive emotional words, and this could be contributed to the delayed access to the emotion value of words relative to facial expressions [131, 147]. Other researches regarding the emotion processing also illustrated the evidence for modulation of the EPN component by facial expressions [65, 148], emotional pictures [64, 149], and emotional words [122, 150, 151]. Combined with the findings that angry faces elicit larger EPN components than happy facial expressions [148, 152], it is therefore concluded that the EPN occurring after N170 may discriminate the discrete emotions.

### 3.4. P300 and LPP (or as LPC)

P300 is a positive-going deflection detected at parieto-occipital electrodes, with sthe peak latency after 300 ms. The P300 discussed here refers to the component extracted from the 300–450 segmentation rather than P300 family. This component has been referred to as indicating the high order cognitive operations associated with attention performance, which may respond to the attentional allocation towards the motivationally salient stimuli. Consistent with such theoretical considerations that the P300 is also sensitive to the stimuli of emotional salience, the early studies regarding emotion processing primarily focus on the P300 effect for emotional valence and arousal and found the significant modulation of P300 amplitudes by the two dimensions [24, 141, 153–155]. For discrete emotions, the recent studies incorporating the P300 analysis also obtained promising results. In a study by Luo et al., the P300 amplitudes for facial expressions showed difference between the fearful and the happy as well as the emotional faces and neutral faces [25], which to some degree extended the previous findings that angry faces elicited larger P300 than happy and neutral faces [156]. Schupp et al. attributed the P300 difference between anger and happy to a more arousal for anger than happy. By contrast, the study by Luo et al. controlled the level of arousal for emotional faces, which would lead to a more convincing conclusion. As we all know, the P300 can be divided into two subcomponents, namely, P3a and P3b. The recent study, respectively, analyzed the two subcomponent amplitudes responding to angry and happy faces and suggested opposite patterns towards emotionality, in which angry elicited smaller P3a amplitudes than happy and larger P3b amplitudes for angry than for happy [144]. The findings about P3b additionally received the support from a subsequent study [143]. This may be due to the less attention orienting towards happy than angry [157]. In a word, P300 can be a valuable tool to explore emotion processing, especially for relationship between emotion and attention.

LPP, also known as LPC, is a positive-going ERP component peaking as early as 300 ms and is generally detected at parietal sites lasting for hundreds of milliseconds after stimulus presentation. LPP is evident at central and frontal midline sites as compared to the earlier parietal positivities, and the component could even persist for several seconds [158, 159]. Especially in studies regarding emotion processing, the PCA analytical method revealed that the LPP significantly differed from the early parietal activities (the activities could be indexed by P300) responding to emotional stimuli [160]. The emotion effect on LPP has received ample supporting evidence from the existent studies. The recent study performed by Zhang et al. verified markedly the modulation of LPC by emotional adjectives, reflecting the larger LPC amplitudes elicited by positive words than the ones by negative words and neutral words [116]. The results are consistent with the previous studies and a subsequent study using emotional nouns [131, 150, 161–163]. For emotional faces, Calvo et al. equally analyzed the LPP response to emotional faces, but the emotion modulation of the component was only seen in upper face half condition with an augmented LPP amplitudes for angry faces than those for the other expressions (neutral, happy, and surprise) [146]. The results supplied a further support for the previous findings regarding the relationship between emotion processing and LPP and additionally to some extent are supported by a subsequent study with RSVP involved [131, 134, 147, 164]. Another ERP study employed the dynamical emotional faces which constituted emotional clips and also found the significant LPC difference among fear, anger, surprise, disgust, sad, and neutral (fear > the rest; anger and surprise > happy) [165]. Combined with the above review, it could be concluded that LPP, mirroring the more elaborative processing of emotion than early and midlatency components, is sensitive to discriminate the discrete emotions.

## 4. From Developmental Emotion and Mental Disorders to Neural Plasticity

In the above section, we reviewed recent decade's progresses in emotion processing investigated by ERP, which indicated the temporal characteristics of emotion processing described by ERP components. On the other hand, emotions cannot be treated as a static competence, but of dynamical developmental properties which are usually accompanied with development of sociality. The development of emotion processing could lead to more appropriate emotional experience and response and also could lead to dysfunctions of emotion processes (like mood disorders), which may be reflected through changes of neural plasticity. Thus, in the following parts, the studies regarding the emotion development and mood disorders will be collected and reviewed, which future researches might put their emphasis on.

### 4.1. Emotion Development and Neural Plasticity

As mentioned in the Introduction, emotion of developmental feasibility could be enhanced through learning (or training) and growing ages or suffers from deficits due to malfunctions of emotion-related neural basis and alternatively disturbances in environmental factors [166, 167]. Researches have investigated these developmental changes of affective processing, by means of behavioral, psychophysical, and neural imaging (fMRI) methods, in humans including healthy normal and patients with mental disorders. Even in adulthood, the emotional competence can be boosted through intervention [168]. And these researches have in turn identified attainable development-dependent changes of emotion processes and cerebral regions that develop along with the growing age or after training. These region changes include greater medial prefrontal control over negative inputs, increased activations in medial orbitofrontal cortex, and putamen for empathy [40, 169]. ERP, despite its incapability to localize the brain regions of developmental changes, could also be employed to explore the developments of emotional processing, which offers an effective instrument for evaluating the changes of processing course of emotion. In the study by Williams et al., advancing age was found to be associated with the decrease of early ERPs over the medial frontocentral electrodes that are supposed to be responsible for happiness [169]. The above changes in the behavior level and cerebral regions to some extent approve the neural plasticity for emotion processing.

From the perspective of psychopathology, the patients with mental disorders who generally somehow manifest the deficits in affective processing should be the targets of emotional investigation, especially affective and mood disorders, alexithymia, autism spectrum disorders, and schizophrenia. Compared to healthy controls, the patients commonly showed significant discrepancy in emotional processes measured with the ERP method, with the detailed results that for instance schizophrenics both suffered disruption of early processes (P100, N170, and N250) and late emotional processing (P300 and LPP) modulated by attention [170–173] and that P100 and P200 for depression were higher evoked by identical sad-eliciting stimuli than for normal people and larger P200 for neutral expressions relative to the positive ones might suggest the diminished perceptual processing of emotion information [174, 175]. These comparative studies between the mental disorders and the healthy could provide exploration of emotion mechanism with the potent and diversified evidences that deficits in emotion processing possibly suggest the disruption of essential mechanism for emotion, which the analysis of localizing the disruption mirrored by ERP discrepancies would serve beneficially to. It is hence critical to emphasize and integrate both emotional findings about normal populations and psychiatric ones.

Unlike organic diseases, mental disorders cannot be attributed to the specific pathogenesis. Especially, for mood disorders of emotion deficits, it meets mass difficulties in locating specifically consistent heterogeneous brain lesions across countless studies, from which the steady and efficient biomarkers could not be easily defined. It is commonly acknowledged nowadays that mood disorders result from the combined influences of physiological and environmental factors (including psychological and social factors). Consistent with such consideration, mood disorders have been attributed to the disturbances in neural plasticity [44]. The neural plasticity refers to a fundamental attribute of nervous systems, the structures and dynamics of which could be altered corresponding to continuing inputs into the systems. And this neural property hence underlies learning. Neural plasticity is a double-edged sword which would lead to the enhancement of related functions owing to the accommodative inputs or would lead to the functional deficits due to the inputs of maladjustments. And such property possibly causes malfunctional psychosomatical symptoms that are behaved by patients with mental disorders, like mood disorders and schizophrenias [44].

Neural plasticity can be assessed by longitudinal studies using fMRI and other techniques, which is an indirect means to investigate the changes of cerebral processing and related regions. Neural plasticity additionally can be measured by LTP and long-term depression (LTD) methods [176], which have been effectively used in animal experiments [177]. And subsequent researchers successfully applied the ERP technique, in which stimulus-specific paradigm (SSP) was employed, to induce the LTP [42]. Other than the SSP method, the ERP component, namely, mismatch negativity (MMN), was confirmed with the capability to index the neural plasticity [178]. On the other hand, N100 repetition reflecting neural adaption also was utilized to investigate the health of plasticity mechanisms required for normal development [179]. Plasticity changes in humans can hence be detected using the noninvasive means of the ERP technique. There exist studies using this method to assess the impaired plasticity of visual and auditory systems in patients with schizophrenia and obtained the significant results [45, 180]. However, seldom studies were reported with the application into emotion processing and patients with mood disorders. Still, we suppose that this plasticity-assessing method with an appropriate improvement might have the potential in assessing the neural plasticity in mood disorders as well. For further study of emotion-related neural plasticity in mood disorders, it is necessary to review the recent progress from the studies regarding emotion processing and neural plasticity in mood disorders, which is expected to offer enlightening for the pathogenesis of mood disorders and offer the potential targets for therapy.

In the following section, we reviewed the emotional studies which emphasized the developmental properties of emotion processing and the studies that focused on mood disorders suffering emotional deficits. Then the neural plasticity for emotion processing was summarized.

### 4.2. Normal versus Mood Disorders

William et al. administered a test to 1000 healthy individuals whose ages cover the lifespan period from 6 to 91 and assessed the explicit as well as implicit recognition of facial emotions, which obtained the result that performance in recognition of emotions improved from childhood through adolescence to early adulthood and then declined in later adulthood [181]. This study provides a powerful evidence for emotion development along with advancing age. Researchers employed the ERP method to investigate the relationship between emotion processing and ages, with the offer of electrophysiological evidence for developing emotion processes. In the study by Kisley et al. [182], the subjects between 18 and 81 years old were asked to categorize the images as positive, negative, or neutral emotion and were simultaneously recorded with EEG device for ERP signals. And the study verified the significant variation of LPP amplitudes with advancing age, in which the LPP amplitudes elicited by negative stimuli were reversely significantly correlated with age while the amplitudes of LPP elicited by positive and neutral ones were not. Such findings regarding the LPP component to some extent reflected the development of top-down regulation for emotion response/experience. Another study performed by Wieser et al. investigated the EPN changes responding to the arousal of emotional stimuli along with the growing age, in which EPN was slightly delayed in elder subjects relative to the younger subjects [183]. The reduction in emotional modulation of the EPN component observed in elderly subjects probably indicates an age-related delay of early visual emotion discrimination. However, Wieser et al. emphasized no influence of this delay on further evaluative processing of emotional stimuli. Combined with the abovementioned study, it could be concluded that the speed of emotion discrimination might decrease from younger adulthood to elderly ones but increased the top-down modulation of emotion response/experience with increasing age to the elderly.

Through training, the process of emotional information gets impacted as well. Especially, in musical training, with accumulated evidences from EEG and fMRI, its capability in changing brain neural plasticity has been verified (for review, see Herholz et al. [184]). ERP as a means also can be employed to assess the plasticity changes related with training. Pinheiro and colleagues compared musicians and nonmusician healthy controls when they listened to sentences with two conditions: sematic content (neutral versus happy versus angry) and pure prosody. The results showed that musicians differed in P200 amplitudes between two conditions, whereas the P200 amplitudes of nonmusicians remained unchanged. And the participants with musical training were more accurate in recognizing angry prosody involved in the sematic sentences [185]. Not only musical training but also mental training, such as meditation, affects significantly neural plasticity and brain functions as well [186]. The ERP study exploring the effects of meditation on emotional processes also offers the evidence for emotional development, in which meditators are less affected by negative stimuli and the influence of positive ones remains unchanged [187]. The above electrophysiological variations with advancing age mirrored the plasticity for neural basis underlying emotion processing, which has been approved by fMRI studies [169, 188].

As mentioned above, the patients with mood disorders, like depression, showed main symptoms manifested in affective deficits mirrored by ERP components compared to healthy controls [189]. And the causes of such disorders have been availably explained towards the damage in neural plasticity, which enables the incapability to harmonically coordinate with experimental inputs, vice versa [190, 191]. A very recent multicenter study enrolled 3036 participants and found the significant association between childhood adversity and subcortical abnormalities, in which increased exposure to childhood adversity led to the smaller caudate volumes in females [192]. It could legitimately be deduced that exactly the subcortical areas of neural plasticity, as a double-edged sword, give rise to the abnormality-orienting changes. Studies using the ERP method found the high-risk individuals (high risk for mood disorders) showing abnormalities in ERP components responding to threatening and negative stimuli compared to the age- and gender-matched low-risk individuals. In studies by Nelson et al. and Kujawa et al., the offspring of depressed parents relative to the offspring of healthy parents demonstrated decreased LPP amplitudes in response to negative and threatening faces and threatening scenes [193, 194]. This discrepancy in emotional response may be due to the dysfunctional plasticity that was inherited from parents of mood disorders [195]. And the neural plasticity additionally is embodied in the invertible properties of such changes. Hetzel et al. assessed the astroglial protein S100B and visually evoked ERPs before and after antidepressant treatment, in which S100B concentration increased as well as that P300 latency was normalized and P200 latency significantly decreased after weeks of treatment [196]. The results are consistent with the subsequent studies using the ERP, MEG, and fMRI methods [197–199].

Neural plasticity, other than the abovementioned cross-sectional and longitudinal studies, could be assessed by LTP or LTD induced by SSP involved in ERP design and could even be assessed by the MMN component evoked in auditory cortex. But unlike the application of LTP into schizophrenia plasticity, few studies were seen to investigate the neural plasticity in mood disorders. As evidenced by the previous studies [42, 43], LTP measured by the ERP technique could be employed to evaluate the neural plasticity of visual and auditory cortex. From this perspective, researchers at least could explore the plasticity of perceptual processing for visual and auditory emotional information through such means. It is therefore noteworthy that future studies regarding dysfunctional neural plasticity associated with mood disorders could consider the practical feasibility of employing the noninvasive ERP technique, which could be combined with PCA and LORETA analytical methods, to define the sources of potential neural plasticity.

## 5. Conclusion

ERP, as a high temporal resolution method, has demonstrated its advantage, compared with the fMRI method, in exploring the temporal dynamics of cerebral activities. Emotion is a complex systematical behavior consisting of peripheral expressions and internal experiences. Compared with its cerebral spatial localization obtaining extremely high-speed progress, the temporal course of emotion processing remains unclear. It is therefore requisite and urgent to push the demand for temporally investigating the mechanism of emotion processing, which application of ERP to some extent could fit. And based on the ERP researches, the three-stage model for processing emotional facial expressions has been proposed by Luo et al.

The neural plasticity enables the emotional development along with the advancing age. And the dysfunctions of neural plasticity for emotion processing have been explained as the causes of mood disorders. ERP, in which the SSP is included, can be employed to investigate the neural plasticity of nervous system. However, such method seldom was applicated into mood disorders, which is waited to be explored in future researches.

Nonetheless, plenty of puzzles with respect to emotion processing remain to be resolved. As reviewed above, most of ERP studies about emotion processing employed the emotional picture, facial expressions, and emotional words as stimuli to elicit emotion response/experience. Other stimulus modalities, like voice, body, and gesture, triggering the emotion response/experience, should receive more attention. The time course of the integration of various emotional modalities is still unclear and needs more consideration. Other issues, for instance the temporal mechanism of social emotion beyond basic emotions and development of emotions, are all worth the efforts, which will be helpful for comprehending what emotion is.
